# Reduced Blood *RGS2* Expression in Mild Cognitive Impairment Patients

**DOI:** 10.3389/fnagi.2021.738244

**Published:** 2021-09-29

**Authors:** Elena Milanesi, Catalina Anca Cucos, Jordi A. Matias-Guiu, Gerard Piñol-Ripoll, Gina Manda, Maria Dobre, Antonio Cuadrado

**Affiliations:** ^1^“Victor Babes” National Institute of Pathology, Bucharest, Romania; ^2^Department of Neurology, Instituto de Investigación Sanitaria San Carlos (IdISSC), Hospital Clínico San Carlos, Universidad Complutense, Madrid, Spain; ^3^Unitat Trastons Cognitius, Hospital Universitari Santa Maria-IRBL Leida, Lleida, Spain; ^4^Department of Endocrine Physiology and Nervous System, Instituto de Investigaciones Biomédicas “Alberto Sols” UAM-CSIC, Madrid, Spain; ^5^Faculty of Medicine, Department of Biochemistry, Autonomous University of Madrid, Madrid, Spain; ^6^Neuroscience Section, Instituto de Investigación Sanitaria La Paz (IdiPaz), Madrid, Spain; ^7^Centro de Investigación Biomédica en Red Sobre Enfermedades Neurodegenerativas (CIBERNED), ISCIII, Madrid, Spain

**Keywords:** *RGS2*, Alzheimer, mild cognitive impairment, biomarker, blood

## Abstract

Regulator of G protein signaling 2 (*RGS2*) is a gene involved in neuronal plasticity and synaptic signaling, whose expression in the brain is altered in neuropsychiatric and neurodegenerative disorders. Microarray data from large datasets suggested reduced *RGS2* mRNA levels in the post-mortem brain tissue and blood of Alzheimer’s disease (AD) patients. The results were previously confirmed by quantitative real-time polymerase chain reaction (qRT-PCR) only *ex vivo* in lymphoblastoid cell lines derived from AD patients and controls. In this study, we compared *RGS2* mRNA levels in peripheral blood samples from 69 mild cognitive impairment (MCI) patients to 50 age- and sex-matched non-cognitively impaired controls, out of which 25 patients were monitored at 1 year. We found that *RGS2* was indeed downregulated in the peripheral blood of these patients (FR = −1.60, *p* < 0.001), and despite disease-specific therapy, *RGS2* transcript levels continued to decrease at 1 year. The results suggest that *RGS2* seems to be involved in AD pathology and progression and can be introduced in a panel of blood AD biomarkers.

## Introduction

Mild cognitive impairment (MCI) is a neurological disorder characterized by a slight but noticeable decline in cognitive abilities, with minimal impact on daily living activities. MCI patients have an increased risk of developing Alzheimer’s disease (AD). It was recently shown that 20–40% of MCI individuals will progress to AD-related dementia within 3 years of the initial diagnosis (Grassi et al., 2019).

Receiving an early AD diagnosis is important for providing appropriate treatment to slow the loss of cognitive function and preserve existing functions. Three specific cerebrospinal fluid (CSF) biomarkers that reflect pathologic aberrations in AD brains are currently used for AD diagnosis (Lewczuk et al., 2020). Specifically, the combination of low β-amyloid (1–42), high TAU, and high phospho-TAU in CSF increases diagnostic accuracy, even in the early phase of the disease (Blennow and Zetterberg, 2009). Besides these CSF biomarkers, other established neuroimaging techniques assess aspects of brain neuroanatomy, chemistry, physiology, and pathology are routinely used, such as magnetic resonance imaging (MRI) and positron emission tomography (PET), which uses specific radiotracers to measure changes in metabolic processes and the accumulation of amyloid plaques in the brain.

Although CSF and neuroimaging biomarker modalities offer reasonable diagnostic accuracy, these procedures feature discomforts and feasibility issues for the patients, mainly related to CSF collection by lumbar puncture, an invasive and potentially risky method. Alternatively, MRI and PET scans are less invasive but are costly and require full cooperation from cognitively impaired patients (Wollman et al., 2004). Moreover, exposure to strong magnetic fields during an MRI scan is risky when patients bear implantable cardiac electronic devices, cerebral artery aneurysm clips, or cochlear or magnetic dental implants (Ghadimi and Sapra, 2021).

In this context, blood-based biomarkers could offer advantages over CSF and neuroimaging biomarkers since the procedure is less invasive and costly, and more acceptable to elderly patients (Lewczuk et al., 2018). During the last decade, researchers have been intensely involved in the identification of new AD biomarkers in plasma, serum, human peripheral blood mononuclear cells (PBMCs), and lymphoblastoid cell lines (LCLs) derived from AD patients. However, reliable and sensitive blood biomarkers have not been identified and adopted for routine use in clinical practice. Several putative mRNA biomarkers identified in PBMCs by RNA sequencing and microarray studies have been suggested (Ma et al., 2019; Shigemizu et al., 2020), but a major limitation is their lack of validation in independent cohorts. A previous study conducted on patient-derived LCLs indicated Regulator of G protein signaling 2 (*RGS2*) mRNA levels as a potential biomarker for AD, showing (by a data mining approach) lower *RGS2* mRNA levels in AD patients compared to non-aged-matched controls in several brain regions and blood (Hadar et al., 2016). However, no study comparing whole blood *RGS2* mRNA levels by qRT-PCR in MCI/AD patients and controls has yet been reported.

RGS2 is a protein that belongs to the GTPase-activating proteins (GAPs) family, which attenuates signaling by heterotrimeric G proteins by selectively inhibiting Gqα function (Berman et al., 1996). Several studies suggest that RGS2 has an important role in modulating G-protein-mediated signaling, including signaling by cholinergic receptors (Hadar et al., 2016). Therefore, this protein might be involved in neuronal activation or desensitization processes that are essential for brain adaptation to external inputs (Ingi and Aoki, 2002).

The aim of this study was to extend previous findings indicating downregulated *RGS2* mRNA levels in LCLs, post-mortem brain tissues, and whole blood from AD patients by exploring through qRT-PCR its mRNA levels in the blood of MCI patients and non-cognitively impaired controls (CTRL).

## Materials and Methods

Sixty-nine patients with MCI due to AD and 50 non-cognitively impaired controls (CTRL) were recruited and diagnosed at the Hospital Universitari Santa Maria-IRBL Leida, Lleida, Spain, and at the Department of Neurology of the Hospital Clinico San Carlos of Madrid, Spain, according to the recommendations issued by the National Institute on Aging and Alzheimer’s Association (Albert et al., 2011). Cognitive function was evaluated using the mini-mental state examination (MMSE); thus, an MMSE score was assigned to each individual included in the study (Table 1). MCI due to AD diagnosis was confirmed by the levels of amyloid-beta 1–40 (Aβ1–40), amyloid-beta 1–42 (Aβ1–42), total TAU and Thr181-phosphorylated TAU (pTAU) proteins in CSF assessed by standardized protocols, using cut-off values established by each clinic. For 33 patients, three CSF biomarkers were evaluated using the Innogenetics enzyme-linked immunosorbent assay (ELISA) kit (pathologic values: Aβ1–42 < 600 pg/ml; TAU > 425 pg/ml; pTAU > 65 pg/ml). The MCI patients presented the following values: Aβ1–42 = 487.7 ± 145.7; TAU = 533.3 ± 260.6; pTAU = 84.6 ± 33.0. For the other 36 patients in the investigated cohort, four CSF biomarkers were analyzed using the Lumipulse G600II instrument (normal value: Aβ1–40 = 7,755–16,715 pg/ml; Aβ1–42 = 725–1,777 pg/ml; ratio Aβ1–42/Aβ1–40 = 0.068–0.115; total TAU = 146–410 pg/ml; pTAU = 21.5–59 pg/ml). These patients had the following values: Aβ1–40 = 15,193.7 ± 5,178.3 pg/ml; Aβ1–42 = 1,000.9 ± 422.1 pg/ml; ratio Aβ1–42/Aβ1–40 = 0.068 ± 0.023; TAU = 690.6 ± 285.9 pg/ml; pTAU = 110.4 ± 49.5 pg/ml). In the last cohort, patients presenting ambiguous values of Aβ1–40 and Aβ1–42 were diagnosed based on TAU and pTAU levels. At the moment of the clinical evaluation, most of the patients were not undergoing AD therapy, with only two patients being on memantine. The demographic and clinical data are presented in Table 1. Venous blood was collected in PAXgene tubes (Qiagen) in the morning after an overnight fast, and total RNA was isolated according to the manufacturer’s protocol. A sub-cohort of 25 patients was monitored at baseline (T0) and after one year (T1) of treatment with acetylcholine esterase inhibitors (rivastigmine or donepezil). Reverse transcription of 400 ng RNA was performed using the RT2 First Strand Kit (Qiagen), and *RGS2* mRNA levels were evaluated by qPCR on an ABI-7500 fast instrument (Applied Biosystems) with SYBR green chemistry. The assay used for *RGS2* was designed on NM_002923. *RGS2* expression levels were normalized on the geometric mean of two housekeeping gene transcripts, *HPRT1* (NM_000194) and *RPLP0* (NM_001002), whose stability in whole blood has been previously assessed (Milanesi et al., 2021), and the statistical analysis was performed on 2^−ΔCt^ values (Supplementary Table). The study was approved by the local ethics committees (Hospital Arnau de Vilanova de Lleida, Lleida, Spain—CE 1218 and Hospital Clinico San Carlos—19/141), and all the participants and/or their caregivers provided written informed consent.

**Table 1:** Demographic and clinical characteristics of the individuals included in the study.

	MCI (*N* = 69)	CTRL (*N* = 50)	Significance
Age	75.6 ± 4.73	73.5 ± 7.52	*p* = 0.075
Sex (%F)	39.13%	54%	*χ*^2^ = 2.586; *p* = 0.108
Education (years)*	9.4 ± 4.15	9.4 ± 4.15	*p* = 0.934
MMSE	24.1 ± 3.21	29.0 ± 1.25	*p* < 0.001
Depression (% affected)	21.7%	16%	*χ*^2^ = 0.612; *p* = 0.434
Anxiety (% affected)	15.9%	22%	*χ*^2^ = 0.706; *p* = 0.401
Hypertension (% affected)	55.1%	40%	*χ*^2^ = 2.636; *p* = 0.104
Cardiopathy (% affected)	11.6%	22%	*χ*^2^ = 2.340; *p* = 0.126
Ictus (% affected)	4.3%	0%	*χ*^2^ = 2.230; *p* = 0.135
Diabetes (% affected)	17.4%	20%	*χ*^2^ = 0.131 ; *p* = 0.717
Hypercholesterolemia (% affected)	49.3%	26%	*χ*^2^ = 6.572; *p* = 0.010
Hypertriglyceridemia (% affected)	0%	2%	*χ*^2^ = 1.392; *p* = 0.238

*Not available for five MCI patients. Differences between mild cognitive impairment patients (MCI) and non-cognitively impaired controls (CTRL) were evaluated using the *t*-test for continuous variables and the *χ*^2^ test for categorical variables.

## Results

**Figure 1:**
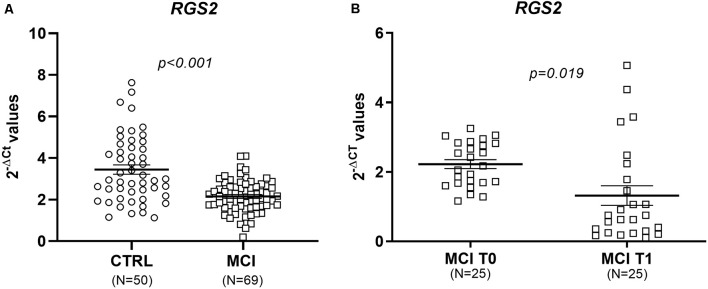
**(A)** Reduced blood *RGS2* mRNA levels in mild cognitive impairment (MCI) patients *vs*. non-cognitively impaired controls (CTRL); (FR = −1.60). Data are presented as 2^−ΔCt^ values and bars represent the expression averages ± standard error of mean (SEM). The *p*-value was calculated using the Mann–Whitney U test. **(B)** Blood *RGS2* mRNA levels in the sub-cohort of 25 MCI patients evaluated at baseline (T0) and after one year (T1) of therapy with rivastigmine or donepezil. Data are presented as 2^−ΔCt^ values and bars represent the expression average ± SEM. The *p*-value was calculated using the related samples Wilcoxon signed rank test.

**Figure 2:**
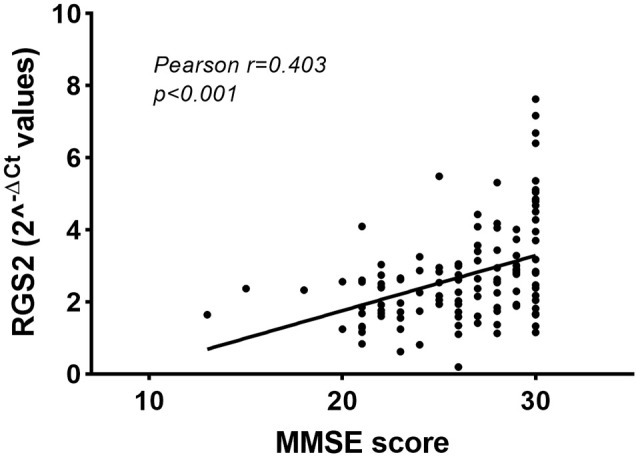
Correlation between blood *RGS2* mRNA levels and mini-mental state examination (MMSE) scores.

**Figure 3:**
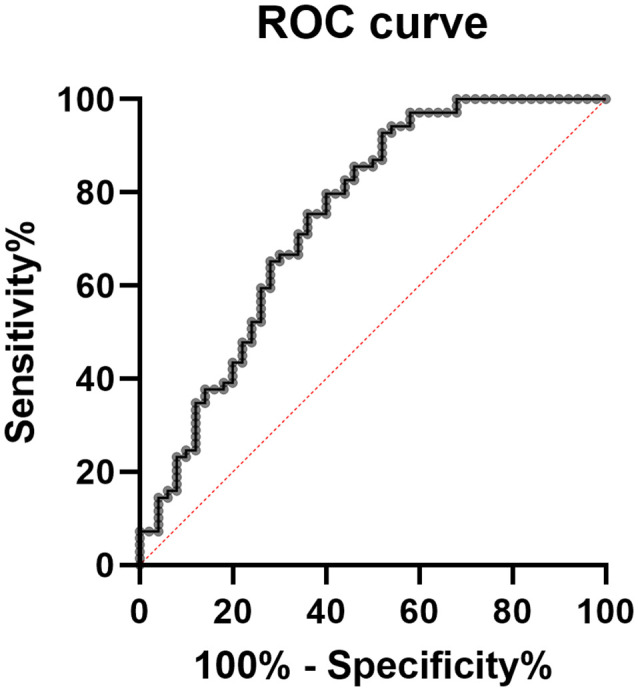
Receiver operating characteristic curve (ROC) based on blood *RGS2* mRNA levels; area under the curve (AUC) = 0.746.

The two groups of patients and CTRL were homogenous for sex, age, education level, and presence of comorbidities, except for hypercholesterolemia, which was more frequent in the MCI group (Table 1). We first compared the *RGS2* levels between individuals with and without hypercholesterolemia, and no significant differences were observed (*p* = 0.427). We further correlated *RSG2* mRNA levels with age and, no correlation was observed in the whole group (*p* = 0.897; *r* = −0.012), in MCI patients (*p* = 0.235; *r* = 0.145), or in CTRL (*p* = 0.692; *r* = 0.057), highlighting that the changes in *RGS2* levels are not age-dependent. We also correlated *RSG2* mRNA levels with CSF biomarkers and no significant correlation was found for the 33 patients evaluated with the Innogenetics ELISA kit (Aβ1–42: *p* = 0.551, Pearson *r* = −0.115; TAU: *p* = 0.984, Pearson *r* = −0.004; pTAU: *p* = 0.533, Pearson *r* = −0.121), nor for the other 36 patients investigated using the Lumipulse G600II instrument (Aβ1–40: *p* = 0.297, Pearson *r* = 0.181; Aβ1–42: *p* = 0.728, Pearson *r* = 0.061; TAU: *p* = 0.936, Pearson *r* = 0.014; pTAU: *p* = 0.832, Pearson *r* = −0.037). Since *RGS2* mRNA levels were not normally distributed (Kolmogorov–Smirnov, test *p* < 0.05), the differences between the MCI and CTRL groups were evaluated with the Mann–Whitney non-parametric test. We found that *RGS2* levels were downregulated in the blood of MCI patients compared to controls (FR = −1.60, *p* < 0.001; Figure 1A). A slight positive correlation between the MMSE score and *RGS2* mRNA levels was observed (*p* < 0.001 and Pearson *r* = 0.403; Figure 2). The receiver operating characteristic (ROC) curve was created, and the area under the curve (AUC) was calculated to assess the potential value of the *RGS2* transcript in discriminating MCI patients from controls. We found an AUC value of 0.746, indicating a fair prediction of the disease (Figure 3). In the sub-cohort of 25 MCI patients evaluated after one year (T1) of therapy with acetylcholine esterase inhibitors (rivastigmine or donepezil) the *RGS2* mRNA levels significantly decreased compared to baseline levels (T0 = 2.22 ± 0.637; T1 = 1.319 ± 1.419; *p* = 0.019, according to Wilcoxon signed rank test). In particular, for 21 patients out of 25 a decrease of 66 ± 25% was registered (Figure 1B), in parallel with a small but statistically significant decrease of the MMSE score (MMSE T0 = 23.88 ± 2.18; MMSE T1 = 22.36 ± 2.95; *p* = 0.004, according to the Wilcoxon signed rank test), showing that the levels of *RGS2* continued to decrease despite the treatment.

## Discussion

Our qRT-PCR study detected a 1.6-fold decrease in the *RGS2* mRNA levels in the whole blood of MCI patients compared to CTRL, supporting previous results obtained from LCLs derived from AD patients, and microarray data from the blood of MCI and AD patients. We also observed a positive correlation between the MMSE score and *RGS2* blood levels, as well as a significant decrease in this transcript during the progression of the disease.

At neuronal synapses, RGS proteins together with G proteins and GPCRs, act in regulating neurotransmitter release, down-stream signaling, synaptic transmission, and synaptic plasticity (Gerber et al., 2016). RGS2, which is highly expressed in the brain, regulates G(i/o) as well as G(q)-coupled receptor pathways. *RSG2* is an immediate-early gene, whose expression is rapidly induced in the neurons of the hippocampus, cortex, and striatum in response to stimuli that evoke plasticity (Ingi et al., 1998). In hippocampal neurons, in particular, RGS2 was shown to increase synaptic vesicle release by downregulating the G(i/o)-mediated presynaptic Ca(2+) channel inhibition, determining short-term synaptic plasticity (Han et al., 2006). RGS2 also has a role in postsynaptic spines in the context of long-term synaptic plasticity (Gerber et al., 2016).

Genetic variants within the *RGS2* gene have been associated with different neuropsychiatric diseases such as anxiety disorders across multiple levels of assessment (childhood temperament, adult personality, and brain function; Smoller et al., 2008; Le-Niculescu et al., 2011; Gottschalk and Domschke, 2017) and panic disorder (Hohoff et al., 2015). Of note, the 3’ UTR single nucleotide polymorphism rs4606 in *RGS2* was associated with post-traumatic stress disorder under conditions of lifetime exposure to a potentially traumatic event and low social support (Amstadter et al., 2009a), suicidal ideation (Amstadter et al., 2009b), and depressive disorders after childhood adversity (Asselmann et al., 2018). Moreover, individuals carrying specific *RGS2* polymorphic variants may experience differential affective responses to smoking tobacco, which could make them vulnerable to developing nicotine addiction (Rorabaugh et al., 2018). Other studies suggested that polymorphic loci in *RGS2* seem to predict the severity of schizophrenia symptoms (Campbell et al., 2008), or they can be associated with the risk of extrapyramidal symptoms induced by typical neuroleptics-haloperidol (Gareeva et al., 2013). Recently, *RGS2* has also been associated with attention deficit hyperactivity disorder (ADHD; McCaffrey et al., 2020).

Regarding neurodegenerative diseases, decreased striatal *RGS2* expression was found in Huntington’s disease patients (Seredenina et al., 2011), and the observed altered expression of *RGS2* mRNA levels in the striatum of rats undergoing dopamine depletion suggested a role in Parkinson’s disease (PD; Geurts et al., 2003). *RGS2* has been indicated as a promising target for interfering with neurodegeneration due to LRRK2 mutations in PD patients (Dusonchet et al., 2014). Moreover, associations between *RGS2* polymorphisms and antipsychotic-induced Parkinsonism have been evidenced (Greenbaum et al., 2009; Higa et al., 2010).

A single study investigating the role of *RGS2* in AD has been conducted thus far, showing that *RGS2* mRNA levels have a 3.3-fold lower expression in AD LCLs compared with controls. Moreover, lower *RGS2* expression levels were found in public gene expression datasets from post-mortem MCI and AD brain tissues compared with controls in the posterior cingulate, superior frontal gyrus, medial temporal gyrus, and in the blood (Hadar et al., 2016).

In conclusion, our results evidenced decreased *RGS2* mRNA levels in the blood of MCI patients, as compared to controls, confirming and validating previous findings through qRT-PCR (Hadar et al., 2016). Moreover, we found that *RGS2* blood levels decreased during disease progression. Altogether, our study suggests that *RGS2* might be a valuable blood biomarker in AD that has the potential to be implemented for clinical use if included in a panel of multiple biomarkers.

## Data Availability Statement

The original contributions presented in the study are included in the article/Supplementary Material, further inquiries can be directed to the corresponding author.

## Ethics Statement

The studies involving human participants were reviewed and approved by Hospital Arnau de Vilanova de Lleida, Lleida, Spain—CE 1218 and Hospital Clinico San Carlos—19/141 ethics committees. The patients/participants provided their written informed consent to participate in this study.

## Author Contributions

EM designed and coordinated the study, performed the statistical analysis, and wrote the first draft of the manuscript. GP-R and JM-G were responsible of the clinical evaluation and the samples collection. MD and CC performed the laboratory experiments, contributed to data processing, and writing. GM and AC gave their contribution in data interpretation, writing and critical reading, and revision. All authors contributed to the article and approved the submitted version.

## Conflict of Interest

The authors declare that the research was conducted in the absence of any commercial or financial relationships that could be construed as a potential conflict of interest.

## Publisher’s Note

All claims expressed in this article are solely those of the authors and do not necessarily represent those of their affiliated organizations, or those of the publisher, the editors and the reviewers. Any product that may be evaluated in this article, or claim that may be made by its manufacturer, is not guaranteed or endorsed by the publisher.
